# Genome-Wide Identification and Expression Analysis of *CCO* Gene Family in *Liriodendron chinense*

**DOI:** 10.3390/plants12101975

**Published:** 2023-05-14

**Authors:** Guoxia Xue, Lingfeng Hu, Liming Zhu, Ya Chen, Chen Qiu, Ruifang Fan, Xiaoxiao Ma, Zijian Cao, Jinhui Chen, Jisen Shi, Zhaodong Hao

**Affiliations:** Key Laboratory of Forest Genetics & Biotechnology of Ministry of Education, Co-Innovation Center for Sustainable Forestry in Southern China, Nanjing Forestry University, Nanjing 210037, China

**Keywords:** *CCO* gene family, *L. chinense*, drought stress

## Abstract

Carotenoid cleavage oxygenase (CCO) is an enzyme that can catalyze carotenoids to volatile aromatic substances and participate in the biosynthesis of two important phytohormones, i.e., abscisic acid (ABA) and strigolactone (SL). However, the genome-wide identification and analysis of the *CCO* gene family in the rare and endangered woody plant *Liriodendron chinense* has not been reported. Here, we performed a genome-wide analysis of the *CCO* gene family in the *L. chinense* genome and examined its expression pattern during different developmental processes and in response to various abiotic stresses. A total of 10 *LcCCO* genes were identified and divided into 6 subfamilies according to the phylogenetic analysis. Subcellular localization prediction showed that most of the LcCCO proteins were located in the cytoplasm. Gene replication analysis showed that segmental and tandem duplication contributed to the expansion of this gene family in the *L. chinense* genome. *Cis*-element prediction showed that *cis*-elements related to plant hormones, stress and light response were widely distributed in the promoter regions of *LcCCO* genes. Gene expression profile analysis showed that *LcNCED3b* was extensively involved in somatic embryogenesis, especially the somatic embryo maturation, as well as in response to heat and cold stress in leaves. Furthermore, qRT-PCR analysis showed that *LcNCED3b* obviously responded to drought stress in roots and leaves. This study provides a comprehensive overview of the *LcCCO* gene family and a potential gene target for the optimization of the somatic embryogenesis system and resistance breeding in the valuable forest tree *L. chinense*.

## 1. Introduction

Carotenoids are generally a kind of isoprenoid compound, which are widely found in all phototrophs and many heterotrophs [1]. Higher plants, algae, fungi and bacteria have the function of carotenoid biosynthesis, while animals cannot produce carotenoids autonomously but can obtain them through food intake [2]. According to their different structural characteristics at the chemical level, carotenoids can be divided into two main organic compounds, namely lutein and carotenoid [3]. Carotenoids play a series of important biological functions in plants, which affect their growth and development. For example, carotenoids, as photosynthetic auxiliary pigments, assume a vital role in photosynthesis in many plants, contributing to processes such as light absorption, electron transfer and oxygen free radical removal, which can protect chlorophyll from photooxidation damage and protect membrane lipids and membrane proteins [4,5]. Carotenoids in plant cells are biosynthesized and stored in plastids, and their colors are red, orange and yellow [6], which are the main pigment factors in the color characters of flowers, leaves and fruits of plants [7,8]. In addition, carotenoids are cleaved to generate various natural small molecules related to hormones, volatiles and signal transduction. For example, the synthetic precursors of some plant hormones such as abscisic acid (ABA) and strigolactones (SL) are carotenoids, which have a crucial function in plant growth, development and signal transduction [9].

The homeostasis of carotenoids hinges on the metabolic homeostasis between carotenoid biosynthesis and degradation [10]. In plants, carotenoid cleavage oxygenases (CCOs) are a class of dioxygenase enzymes that perform catalytic functions in the cleavage of carotenoids and their conjugate double bonds. The catalytic activity of CCOs is the key to regulating carotenoid accumulation. Carotene (β-carotene) and its downstream carotenoids could be degraded and produce ABA, SL and other volatile compounds [11,12,13,14]. It is one of the major factors that contributes to carotenoid degradation in plants.

Previous studies have shown that the genes that encode the CCO proteins in plants are a small family of two subfamilies, which can be divided into two subfamilies, namely CCD and NCED [15], and all CCO proteins have a conserved domain called retinal pigment epithelium membrane protein (RPE65) [16]. The first *CCO* family member was *VP14*, a member of the *NCED* subfamily, isolated from maize, which provided an important basis for exploring *CCO* genes in other species [17]. Subsequently, based on *VP14* homology analysis, a variety of *CCO* family members involved in the oxidative cracking of carotenoids were identified. With the development of genomics and bioinformatics, the *CCO* family genes have been identified in many plants, such as *Malus domestica* [18] (21), *Vitis vinifera* [19] (19), *Cucumis melo* [20] (9), *Populus trichocarpa* [21] (23), *Litchi chinensis* [22] (15), etc. In the model plant *Arabidopsis thaliana*, the *CCO* family contained nine members, of which the *CCD* subfamily had four genes (*CCD1*, *CCD4*, *CCD7* and *CCD8*) and the *NCED* subfamily included five genes (*NCED2*, *NCED3*, *NCED5*, *NCED6* and *NCED9*) [23]. Then, in 2016, researchers identified a new family member of the *CCOs* in *Solanum Lycopersicum* for the first time and named it *CCD*-like (*CCDL*) [24].

Numerous studies have shown that the *CCO* gene family has important biological functions in plants, such as participating in the biosynthesis of aroma volatiles and plant hormones, involving in responses to various abiotic stresses, pigmentation, photosynthesis and light protection. Studies have shown that *CCD1* and *CCD4* can specifically lysate β-carotene into the aromatic volatile carotenoid β-ionone [25], which is one of the main components of the flower aroma in plants. At the same time, the cleavage activity of *CCD4* can significantly reduce the accumulation of β-carotene in the plastids, so it has a significant effect on the flower and fruit color of some plants [26]. In many plants, the *CCD7* and *CCD8* genes in the family have been shown to be synergistically involved in the biosynthesis of the plant hormone aurolactone and in the mediation of plant growth and development [27,28]. The expression regulation of *CCD7* and *CCD8* can further regulate morphogenesis during plant development [29]. For example, *CCD7* and *CCD8* are key genes in the biosynthetic pathway of SL [30,31], and SL is a new sort of plant hormone, which can inhibit plant branching or tillering [32], regulate root development [33] and participate in response to multiple stresses [34,35]. In addition, all *NCED* genes are involved in the biosynthesis of ABA in plants, and *NCEDs* can cleave violaxanthin and neoxanthin into xanthaldehyde, which is the first step in the biosynthesis of ABA in plants and a key rate-limiting step. Subsequently, the xanthaldehyde is transported into the cytoplasm, and after a series of reactions, the plant hormone ABA is finally formed [36].

The genome information of *L. chinense* was decoded for the first time in 2019 [37]. *CCO* is a very important gene family in plants, but there are few reports on the characteristics of *CCO* genes in *L. chinense*. In this study, the genome-wide identification and characterization of the *LcCCO* gene family will be conducted based on the *L. chinense* genome data. The transcriptional expression of *LcCCO* family genes will be analyzed in different organs, somatic embryogenesis and under different stresses, in order to further reveal the function and expression pattern of *LcCCOs* and offer a theoretical basis for the application of *LcCCOs* in breeding research.

## 2. Results

### 2.1. Identification and Characterization of CCO Genes in L. chinense

In this study, 9 CCO protein sequences (*AT1G30100.1*, *AT1G78390.1*, *AT2G44990.1*, *AT3G14440.1*, *AT3G24220.1*, *AT3G63520.1*, *AT4G18350.1*, *AT4G19170.1*, *AT4G32810.1*) of *Arabidopsis thaliana* were used as query sequences to search against the local protein database of *L. chinense*, and then a total of 10 *LcCCO* genes (*Lchi23418*, *Lchi06122*, *Lchi06123*, *Lchi24696*, *Lchi08467*, *Lchi06042*, *Lchi30590*, *Lchi08901*, *Lchi08902*, *Lchi08924*) were identified from *L. chinense* by HMM search and BLASTP, and these sequences can be downloaded from the website (https://ftp.cngb.org/pub/CNSA/data1/CNP0000295/CNS0044063/CNA0002404/, accessed on 11 February 2023). In order to further understand the physicochemical properties of the LcCCO protein, we analyzed the basic characteristics of the gene family members (Table 1), including coding sequence length (CDS), the number of amino acids in the protein, isoelectric point (pI), relative molecular weight (Mr), grand average of hydropathicity (GRAVY), and subcellular localization. The results showed that the CDS length among all *LcCCOs* was 1056 bp to 1869 bp, and the encoded protein size ranged from 351 to 622 aa. The pI index ranged from 5.27 to 7.73, and the lowest MW value was LcCCDLb (39.8 kDa) and the highest was LcCCDLa (70.2 kDa). The GRAVY of 10 LcCCO proteins ranged from −0.299 to −0.151, which indicated that all LcCCOs were hydrophilic proteins. Subcellular localization prediction analysis showed that among the 10 LcCCO proteins, 8 members were located in the cytoplasm, 1 member was located in the chloroplast, and 1 member was predicted to be Pin the chloroplast and cytoplasm.

### 2.2. Phylogenetic Analyses of LcCCO Genes

**Figure 1:**
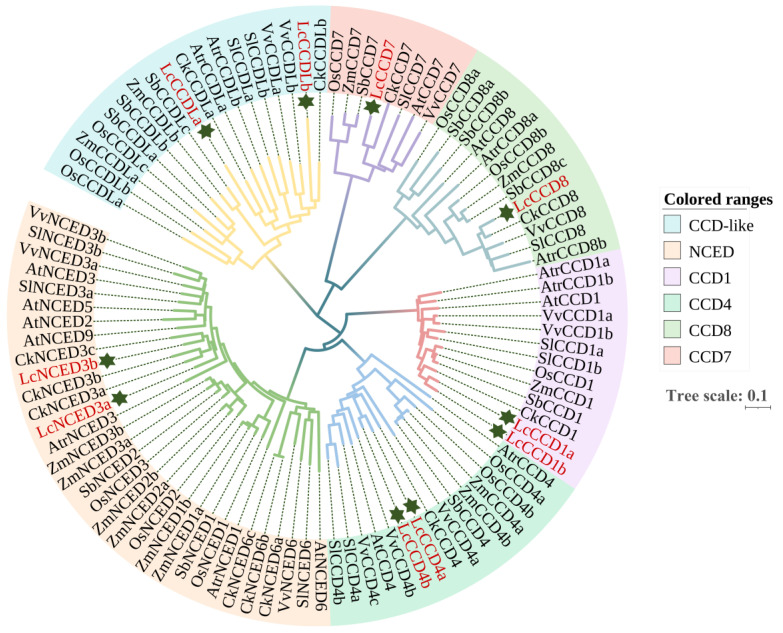
Phylogenetic tree of CCO proteins in nine plant species: *L. chinense* (Lc), *Vitis vinifera* (Vv), *Solanum lycopersicum* (Sl), *Oryza sativa* (Os), *Sorghum bicolor* (Sb), *Zea mays* (Zm), *Cinnamomum kanehirae* (Ck) and *Amborella trichopoda* (Atr). ClustalW program of MEGA-X was used for multiple sequence alignment. MEGA-X was also used to build the neighbor-joining (NJ) tree, and the bootstrap repeat value parameter was set to 1000 times. The visualization of phylogenetic trees is achieved through the online website iTOL. The stars represent CCO family proteins of *L. chinense*. The distance scale “Tree scale 0.1” represents the unit length of the difference values between sequences. The CCO protein/gene IDs of each species are listed in Supplementary Table S1, respectively.

To further investigate the phylogeny of LcCCOs, 99 protein sequences from 9 species, including *L. chinense*, *Arabidopsis thaliana*, *Vitis vinifera*, *Solanum lycopersicum*, *Oryza sativa*, *Sorghum bicolor*, *Zea mays*, *Cinnamomum kanehirae* and *Amborella trichopoda*, were used to build a phylogenetic tree of CCOs in these species (Figure 1). The results indicated that the 99 CCO proteins were divided into 2 sets, including 6 subfamilies (CCD1, CCD4, CCD7, CCD8, NCED and CCD-like (CCDL)), which was consistent with previous phylogenetic analyses of CCO proteins in plants. The volitionary relationship between CCDL and NCED proteins was relatively distant, whereas the relationship between CCD4 and NCED proteins was relatively close. CCD1 (LcCCD1a, LcCCD1b), CCD4 (LcCCD4a, LcCCD4b), CCD7 (LcCCD7), CCD8 (LcCCD8), and NCED (LcNCED3a, LcNCED3b) subfamilies included 2, 2, 1, 1, 2, members of *L. chinense*, respectively. Notably, the CCD-like subfamily, which was identified first in tomato in 2016 [24], contained a minimum of 2 *CCDL* genes in *L. chinense*, *Vitis vinifera*, *Solanum lycopersicum*, *Oryza sativa*, *Sorghum bicolor*, *Zea mays*, *Cinnamomum kanehirae* and *Amborella trichopoda*, whereas none were identified in *Arabidopsis thaliana*. This suggested that *CCDL* genes probably existed and expanded only within some species. In addition, previous results showed that there were five members of the *NCED* gene family in *Arabidopsis thaliana*, namely *NCED2*, *3*, *5*, *6*, and *9*. However, only two members were identified in the *LcNCED3* class and *NCED2*, *5*, *6*, *9* classes were not found in *L. chinense*.

### 2.3. Gene Structure, Conserved Motif Analyses of CCO Genes

**Figure 2:**
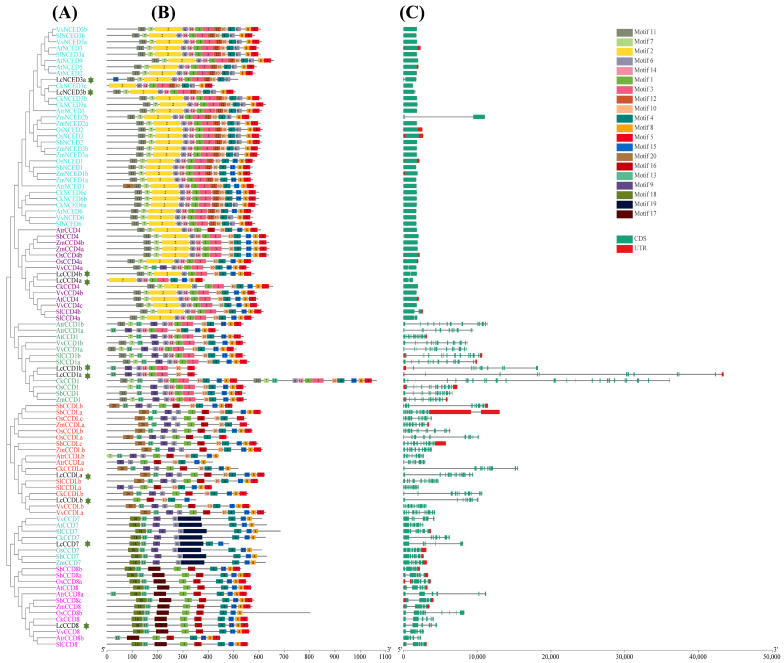
Gene structure, conserved motif analyses of 99 *CCO* genes from *L. chinense*, *Arabidopsis thaliana*, *Vitis vinifera*, *Solanum lycopersicum*, *Oryza sativa*, *Sorghum bicolor*, *Zea mays*, *Cinnamomum kanehirae* and *Amborella trichopoda*. (**A**) Phylogenetic tree of *CCO* genes. (**B**) The conserved motifs of *CCO* proteins were identified by the MEME website and visualized with TBtools. (**C**) Gene structure of *CCOs*. The stars represent *CCO* family genes of *L. chinense*. The conserved motifs and gene structure information of *CCO* genes are organized in Supplementary Table S1.

To further elucidate the gene structure characteristics of *CCO* family genes, the exon-intron structures and conserved motifs composition were analyzed (Figure 2, Table S1). In conservative motif analysis, a total of 20 different motifs with 21-107 amino acid residues were found in the CCO gene family using the MEME tool. In order to accurately grasp the composition of motifs between two groups, NCED and CCD (CCD1, CCD4, CCD7, CCD8 and CCDL), we counted the number of motifs contained in each family member. The statistical results showed that there was a significant difference in the number of motifs between the NCED and CCD groups. In the NCED group, except for AtrNCED1, LcNCED3a, CkNCED3c, and SlNCED3a, the number of motifs was 13, while in the CCD group ranged from 6 to 14, except for the special CkCCD1, which contained 24 motifs. It was further indicated that the difference in the number and type of conserved motifs among different branches of the CCD subfamily were significantly higher than those among different branches of the NCED subfamily. Notably, as is shown in Figure 2, motifs 4, 15, and 8 were widely distributed in the CCO family, accounting for 98%, 72% and 92%, respectively, and they were all located near the C-terminal of these proteins. These results suggested that the three motifs were highly conserved throughout the CCO gene family, which might play a major role in some common biological functions. Combined with phylogenetic tree and motif analysis results, motif distributions of CCOs also displayed different patterns in the different clades. For example, motif 12, motif 20, motif 19 and motif 17 only existed in the NCED, CCDL, CCD7 and CCD8 subfamilies, respectively. In addition, only the CCD7 subfamily lacked motif 1 and motif 5, and the CCD8 subfamily lacked motif 6, which existed in all the other subfamilies. These motifs may be the main factors leading to the differences in gene function between subfamilies. The phylogenetic tree showed that the evolutionary relationship between NCED and CCD4 subfamily proteins was relatively close. Only NCED and CCD4 subfamilies had motif 2 and they lacked motif 13 at the same time, except for VvCCD4a. In addition, both NCED and CCD4 subfamilies had motif 1, motif 3, motif 4, motif 5, motif 6, motif 7, motif 8, motif 10, motif 11, and motif 14, and they lacked motif 16 (except AtrCCD4), motif 17, motif 18, motif 19, motif 20 (except AtrNCED1). These findings indicated that the *CCO* genes with a close evolutionary relationship showed similar motif composition.

By extracting and analyzing the genomic annotation information of 99 CCO genes in the above 9 plant species, we obtained the intron-exon structure information to further explore the structural composition of these genes. Gene structure analysis showed that the exon number of *CCO* genes varied from 1 to 25, and that of *LcCCO* genes ranged from 2 to 13. *CCD1* and *CCDL* subfamilies had the largest number of exons, followed by *CCD7* and *CCD8* subfamilies, while *NCED* and *CCD4* subfamilies only had one or two exons. Interestingly, *NCED* and *CCD4* subfamilies had a similar gene structure and gene length, except for *ZmNCED2b*, which was relatively long. Other subfamilies showed different patterns of gene structure and gene length. Regarding the distribution pattern of exons in the *CCO* gene family, the *CCD1* and *CCDL* subfamilies also contained the largest number of introns, followed by the *CCD7* and *CCD8* subfamilies, while the *NCED* and *CCD4* subfamilies had the least introns. Among 47 members of the *NCED* and *CCD4* subfamily, only 4 genes contained an intron, while the rest had no intron. Overall, even though the exons and introns were in different positions, the gene structure and exon and intron numbers within the same subfamily showed similarities.

### 2.4. Analysis of Cis-Acting Elements of LcCCO Gene Promoters

**Figure 3:**
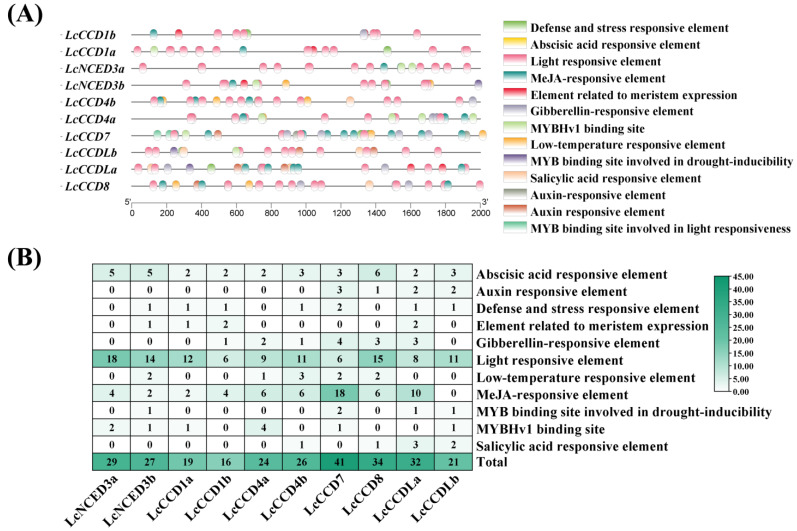
Prediction of *cis*-acting elements of *CCOs* in *L. chinense*. (**A**) Detailed information of *cis*-acting elements on each *LcCCO* gene promoter. (**B**) Heat map showing the number of *cis*-acting elements on the *LcCCO* gene promoters. Details of *cis*-acting elements in *LcCCO* promoters are shown in Supplementary Table S1.

Different *cis*-acting elements in gene promoters might reveal that these genes have corresponding functions in plant growth and development and some stress responses [38]. In order to further explore which biological processes can be regulated by *LcCCOs*, the 2000 bp DNA upstream sequences of the translation initiation site of *LcCCO* genes were obtained from the *L. chinense* genome file. Then, their assumed *cis*-acting elements were predicted through the PlantCARE database. In this study (Figure 3), there were altogether 269 *cis*-acting elements of the three types of promoters, including 120 plant hormone response elements (abscisic acid responsive element, gibberellin responsive element, auxin response element, MeJA responsive element, and salicylic acid responsive element); 128 environment response elements (low-temperature responsive element, defense and stress responsive element, light responsive element); 15 MYB binding sites (MYB binding site involved in drought inducibility, MYBHv1 binding site); and 6 elements related to meristem expression, that were analyzed in all *LcCCO* genes. These results suggested that *LcCCO* family genes might play a role in plant growth and development as well as response to various environmental stresses and hormone signaling.

### 2.5. Expression Patterns Analysis of LcCCO Genes by RNA-Seq

**Figure 4:**
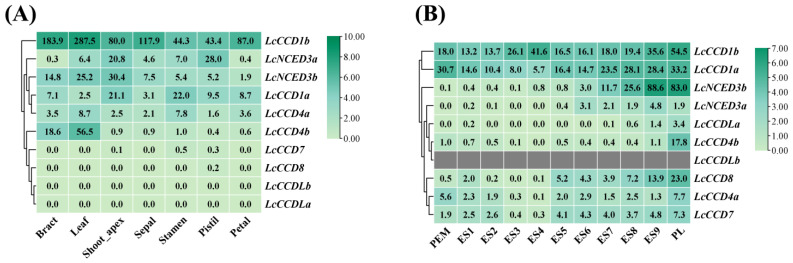
Expression patterns of *LcCCO* genes in different organs and somatic embryogenesis. The heat map shows the mean of biological replicates. (**A**) The expression level of *LcCCO* genes in different organs, including bract, leaf, shoot apex, stamen, petal, pistil, and sepal. (**B**) The expression level of *LcCCO* genes in somatic embryogenesis of hybrid *Liriodendron*. Detailed expression data of related genes are shown in Supplementary Table S2.

To elucidate the role of *LcCCO* family genes in different organs, we analyzed their expression levels in seven organs of *L. chinense* (including bract, leaf, shoot apex, stamen, petal, pistil, and sepal) and mapped their expression heat map using TBtools software (Figure 4A). According to the expression trend of *LcCCO* family genes in different organs, it can be seen that compared with other family members, *LcCCD1b* is highly expressed in seven organs, with the highest expression level in leaves. Furthermore, the *LcCCD1a* was highly expressed in the shoot apex and stamen but was low in the other five organs. The *LcNCED3a* was mainly expressed in the shoot apex and pistil, with low expression in the leaves, sepal, and stamen, and was barely expressed in the bract and petal. The expression of *LcNCED3b* in the bracts, leaves and shoot apex was higher than that in the other four organs. The expression level of *LcCCD4a* was low in all organs, while *LcCCD4b* was specifically high in bracts and leaves but hardly expressed in other organs. In addition, *LcCCD7*, *LcCCD8*, *LcCCDLa* and *LcCCDLb* were not expressed in all organs.

In order to further explore the role of the *CCO* gene in different stages of somatic embryogenesis, transcriptional expression analysis was conducted based on existing transcriptome data (Figure 4B). Transcription analysis showed that *LcCCD1a* and *LcCCD1b* were expressed in all 11 stages of somatic embryogenesis. *LcCCD1b* was down-regulated from PEM to ES2, down-regulated from ES3 to ES4, and stable at ES5-6, and then it was gradually up-regulated again, reaching the highest level in the PL stage. In the whole process, the expression of *LcCCD1a* was first down-regulated and then up-regulated, with the lowest expression level in the ES4 stage and the highest expression level in the PL stage. *LcNCED3b* began to be expressed in ES6, was barely expressed from PEM to ES5, and was mainly expressed from ES8 to PL. The expression level of *LcNCED3b* was the highest in ES9, which was 29.5 times and 7.5 times the levels of ES6 and ES7, respectively. This shows that it might be mainly involved in the late process of somatic embryogenesis. Among the remaining seven gene family members, only *LcCCD4b* and *LcCCD8* had relatively high expression in the PL stage, while other genes were expressed at very low levels or were not expressed in the whole somatic embryogenesis process.

**Figure 5:**
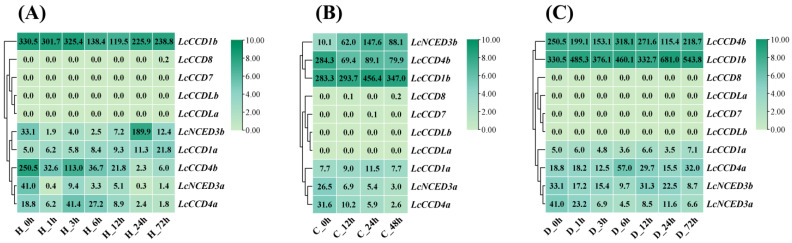
Expression patterns of *LcCCO* genes in leaves of hybrid *Liriodendron* under three stresses. The heat map shows the mean of three or four biological replicates. (**A**) The expression level of *LcCCO* genes under high-temperature (40 °C) stress. (**B**) The expression level of *LcCCO* genes under low-temperature (4 °C) stress. (**C**) The expression level of *LcCCO* genes under drought stress. Detailed expression data of related genes are shown in Supplementary Table S2.

Existing studies have shown that many plant hormones play a crucial role in abiotic stress response, participating in the mediation of plant responses to abiotic stress and protecting plants from the negative effects of abiotic stress [39]. In the analysis of *cis*-acting elements, it was found that there were a major number of elements in the *LcCCO* promoter region responding to stress and various plant hormone signals, which indicated that *LcCCO* genes might be involved in the stress regulation response of plants. Therefore, in order to further explore the role of *LcCCO* family genes in abiotic stress, this study conducted a transcriptional expression analysis of the *LcCCO* gene family based on transcriptome data of hybrid *Liriodendron* plants under high-temperature, low-temperature, and drought stress (Figure 5).

Under heat stress (Figure 5A), compared with other family members, *LcCCD1b* expression was higher in the whole process, first showing a trend of down-regulation and then up-regulation. There was no significant difference in the expression level from 0 h to 3 h, which decreased sharply at 6 h and then increased significantly at 24 h and 72 h. The expressions of *LcCCD4b*, *LcCCD4a* and *LcNCED3a* decreased sharply at the beginning of high-temperature stress and were up-regulated to a certain extent at 3 h, and then they began to down-regulate. Under low-temperature stress (Figure 5B), the expressions of *LcNCED3b* and *LcCCD1b* increased first and then decreased, and they reached the peak at 24 h. Under low-temperature (4 °C) stress, the expressions of *LcNCED3b* and *LcCCD1b* increased first and then decreased, and they reached the peak at 24 h. Compared with the control group, the expression of *LcCCD4b* was down-regulated overall during the treatment process, but there was no significant difference in the expression level at different time periods (12 h, 24 h, 48 h). Under drought treatment (Figure 5C), the expression levels of *LcCCD4b* and *LcCCD1b* fluctuated from 0 h to 72 h with the change of treatment time, and their expression levels were relatively high. The expression of *LcCCD1a*, *LcCCD4a*, *LcNCED3b*, *LcNCED3a* was extremely low at all treatment time points (0 h, 1 h, 3 h, 6 h, 12 h, 24 h and 72 h). It is worth noting that *LcCCD7*, *LcCCD8*, *LcCCDLa* and *LcCCDLb* were not expressed at all in the above three treatments, indicating that these four genes may not be involved in the response to high-temperature, low-temperature, and drought stress and may play a role in other biological processes.

### 2.6. Expression of LcNCED3a/b in Response to Cold and Drought Stress

**Figure 6:**
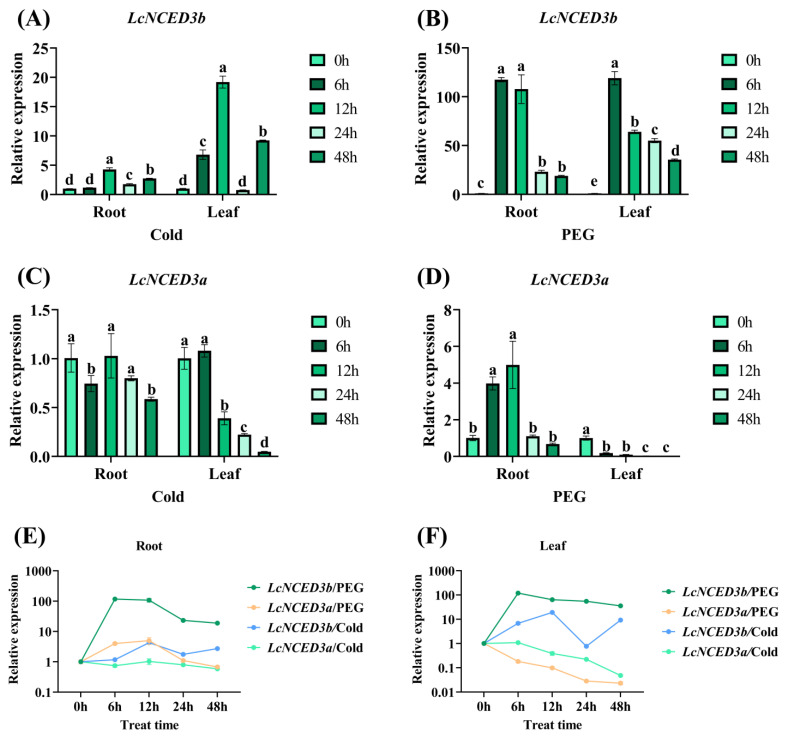
The expression of *LcNCED3a/b* identified by qRT-PCR in roots and leaves under cold and PEG stresses. (**A**–**D**) Expression analysis of *LcNCED3a/b* in different organs under low-temperature and PEG stress. Comparative analysis of *LcNCED3a/b* expression in roots (**E**) and leaves (**F**) under two kinds of stress. Relative expression level of *LcNCED3a/b* was calculated by the 2^−∆∆CT^ method. Different lowercase letters indicate statistically significant differences (ANOVA, 5% level). The vertical bars are representative of SD. All statistics are listed in Supplementary Table S3.

The above transcriptional analysis results showed that *LcNCED3b* responds to both heat and cold stress, but not drought stress, which may be because only leaves were detected in the transcriptional sequencing. In addition, the similarity between *LcNCED3a* and *LcNCED3b* was up to 83%, but the expression level of *LcNCED3a* was very low in all the above treatments. Therefore, in this study, qRT-PCR was used to compare and analyze the expression patterns of *LcNCED3a*/*b* in roots and leaves under cold and drought stress. The results showed that abiotic stress has a significant effect on the expression level of the *LcNCED3b* gene, while *LcNCED3a* was almost not expressed in the control group and the two treatments (Figure 6). Under cold stress, the expression of *LcNCED3b* in both roots and leaves increased first and then decreased, with the highest expression level at 12 h (Figure 6A). Under drought stress, the expression of *LcNCED3b* in roots and leaves increased sharply with time in the early stage and reached a peak at 6 h, and then it began to decrease gradually (Figure 6B). However, the expression of *LcNCED3a* was completely different from that of *LcNCED3b*, which was almost not expressed in cold, drought treatment and in untreated roots and leaves (Figure 6). In addition, we compared the expression of *LcNCED3a/b* in roots and leaves under these two stresses, respectively (Figure 6E,F). As can be seen from the figure, the relative expression levels of *LcNCED3b* in roots and leaves under drought stress were significantly higher than those under cold stress, while the expression level of *LcNCED3a* was very low and had no significant change. The results showed that *LcNCED3b* expression was more affected by drought than cold stress in roots and leaves.

## 3. Discussion

In the carotenoid metabolism pathway, carotenoid cleavage oxygenase (CCO) family members can oxidize and cleave carotenoids at one or both ends of the molecule to form various apocarotenoids, which as the main components of plant hormones, pigments, flavors, flavors and defense substances, have important biological significance in the growth-and development of plants. Hormones such as ABA and SL and non-volatile substances such as crocetin and bixin are all associated with apocarotenoids [40,41]. The CCO proteins catalyze the cleavage of carotenoids and help regulate plant responses to stress [42]. The genes that encode the CCO proteins are from a small gene family that has a small number of members in plants [43]. In general, the CCO gene family is present in most eukaryotes, particularly in various plants. For example, 9, 21, 19, 9, 23 and 15 *CCO* genes were identified in *Arabidopsis thaliana* [23], *Malus domestica* [18], *Vitis vinifera* [19], *Cucumis melo* [20], *Populus trichocarpa* [21], *Litchi chinensis* [22]. These *CCO* genes are a class of specific enzymes that can catalyze the conjugated double-bond system of carotenoids and apocarotenoids to further generate smaller compounds, which has significant effects on plant growth, development, crop quality and response to various stresses. Therefore, it is very necessary to understand the evolutionary relationship and functional characteristics of plant *CCO* genes.

In this study, 10 *LcCCO* genes were identified from the *L. chinense* genome. Previous reports indicated that the *CCO* gene family in *Arabidopsis thaliana* [23] can be divided into the *CCD* subfamily and *NCED* subfamily and then further divided into 5 subfamilies (*NCED* and *CCD1*, *4*,*7*,*8*). Subsequently, researchers discovered a new group of *CCO* family members called *CCDLs*, but only in some plants [24]. In order to further explore the topological clustering and evolutionary relationship of *CCO* genes in different species, 99 CCO protein sequences from 9 plants were selected to construct a phylogenetic tree (Figure 1). According to the phylogenetic tree, 99 *CCO* genes were divided into 6 groups, including *CCD1*/*4*/*7*/*8*, *NCED* and *CCDL*, and the number of genes contained in each group was quite different. In addition, although the clustering of *CCOs* was similar among species, the number of *CCOs* of different species was also different within and between groups, suggesting that *CCO* genes may have expanded to different degrees in different species. According to the clustering of 99 *CCO* sequences in the phylogenetic tree, it was also found that the *NCED* branch was more closely related to *CCD1* and *CCD4*, and was distantly related to *CCD7*, *CCD8* and *CCDL*. Compared with other species, *LcCCO* genes were also clustered into six groups (*CCD1*/*4*/*7*/*8*, *NCED* and *CCDL*), but only *NCED3* was found in the *NCED* subfamily, which may be due to incomplete genomic data, leading to the loss of *NCED2*, *5*, *6* and *9* in *L. chinense*. The analysis results of the physical and chemical properties of proteins show that the molecular characteristics of each LcCCO member are different (Table 1), such as the molecular weight of protein, isoelectric point and GRAVY value. However, they all have a negative GRAVY value, indicating that all 10 LcCCO family proteins are hydrophilic proteins. In addition, the prediction results of subcellular localization showed that nine CCOs were located in the cytoplasm, suggesting that CCO proteins may have important functions in the cytoplasm.

In order to further understand the structural characteristics of *CCO* family genes, we performed a bioinformatics analysis of gene structure and conserved motif composition (Figure 2). The analysis results show that the number and types of motifs contained in the *CCO* protein sequences in the same group were very similar; the groups of *CCO* genes could be easily distinguished according to the motif characteristics in the sequences; and the grouping was consistent with the phylogenetic tree, which proved that the results of the previously constructed phylogenetic tree were credible. Similarly, gene structure analysis also found that the structure of genes in the same group was also highly similar. *CCD1* and *CCDL* groups contained the largest number of exons and introns, while most genes in *NCED* and *CCD4* groups contained only one exon. The similarity of motif and exon constitutive patterns between genes indicates that these genes may have similar functions in some biological processes in plants.

*Cis*-acting element analysis showed that the *LcCCO* promoters contain a large number of hormone response elements (ABA, GA, IAA, MeJA, and SA), stress response elements, and light response elements (Figure 3). Previous studies have shown that many plant hormones not only regulate and affect many physiological processes in plants, but they also play an important role in plant response to stress. For example, SA not only contributes to plant resistance to diseases and insects, but it also plays an important role in plant abiotic stress management. The combination of microorganisms and plants enriched the levels of endogenous SA in plants, thereby inducing systemic resistance and alleviating abiotic stress by inducing SA-mediated signal transduction [44]. As a widespread plant hormone in plants, ABA is involved in a variety of plant growth and development processes, such as promoting seed dormancy and stomatal closure and participating in fruit ripening, etc. However, in more cases, ABA is involved in the anti-stress response of plants as a signal factor of stress, thus participating in the role of plants in resisting adverse environmental stress [45]. Therefore, we not only analyzed the expression of *LcCCOs* in different organs and somatic embryogenesis, but also analyzed the transcriptional expression of *LcCCO* genes under three kinds of stress: high temperature, low temperature and drought.

The transcription analysis of *LcCCOs* in different organs showed that *LcCCD1b* was highly expressed in all seven organs, suggesting that it may have a wide range of functions in the growth, development and morphogenesis of *L. chinense*. LcCCD4b is specifically highly expressed in leaves, suggesting that it may be mainly involved in some physiological processes in leaves. *LcCCD7*, *LcCCD8*, *LcCCDLa* and *LcCCDLb* were not expressed in all organs, suggesting that these genes may not participate in the organ development process of *L. chinense*. The transcription analysis of *LcCCOs* in somatic embryogenesis showed that *LcCCD1a* and *LcCCD1b* were expressed in all 11 stages of somatic embryogenesis, indicating that they may play a functional role in the whole process. *LcNCED3b* was specifically highly expressed in the ES9 and PL stages, indicating that *LcNCED3b* mainly acted in the late stage of somatic embryogenesis.

Stress transcriptional expression analysis showed that the transcriptional expression patterns of *LcCCOs* under three stresses were divided into three types: high expression, low expression, and no expression at all. The details have been clarified above and will not be repeated. It is worth noting that the expression of *LcCCD1b* under high-temperature stress and of *LcCCD1b* and *LcCCD4b* genes under drought stress fluctuates with the extension of treatment time, indicating that these genes may be affected by stress, but whether they play a role in response to stress needs to be further verified. The four genes *LcCCD7*/*8* and *LcCCDLa*/*b* were not expressed in the three stress treatments. Therefore, the specific functions of these genes need to be explored and verified by subsequent designed experiments.

Based on all above transcriptome analysis results, it was found that *LcNCED3b* responded to both heat and cold stress in leaves, but not to drought stress. However, in the qRT-PCR analysis (Figure 6), it was found that the response of *LcNCED3b* was more obvious in leaves than in roots under cold stress, which suggested that *LcNCED3b* might play a major role in leaves under this stress condition. Under drought stress, the relative expression level of *LcNCED3b* changed significantly in both roots and leaves, showing an up-regulated and then down-regulated trend. In the same organs, it was obviously expressed under drought stress, but its relative expression level was very low under cold stress. These results suggested that *LcNCED3b* might be mainly involved in response to drought stress in abiotic stress.

## 4. Materials and Methods

### 4.1. Identification of LcCCO Genes and Analysis of Its Characteristics

In this paper, the genome data file of *L. chinense* was downloaded from the website (https://ftp.cngb.org/pub/CNSA/data1/CNP0000295/CNS0044063/CNA0002404/, accessed on 11 February 2023) to establish a local protein database. The CCO protein sequences of *Arabidopsis thaliana* were obtained from the online database Phytozome v.13 (https://phytozome-next.jgi.doe.gov/, accessed on 11 March 2022) as the queries to search against the local protein database of *L. chinense* with the BLASTP algorithm (e-value = 1 × 10^−5^). Meanwhile, the Hidden Markov Model (HMM) [46] profile (PF03055) of CCO was acquired from the PFAM database (http://pfam.xfam.org/, accessed on 15 March 2022)), which was used as a query to search against the local protein database to obtain protein sequences with the RPE65 domain from the database. We combined the results of both methods and removed redundant sequences to obtain a candidate sequence set. These candidate sequences were further validated based on the domain database: NCBI Conserved Domain Database (https://www.ncbi.nlm.nih.gov/cdd, accessed on 15 March 2022). In the end, all members of the *LcCCO* gene family were identified and named according to the results of the phylogenetic tree.

ExPASy (https://web.expasy.org/protparam/, accessed on 23 May 2022) was used to analyze the physicochemical properties of proteins, including the relative molecular weight (Mr), isoelectric point (pI), number of amino acids and grand average of hydropathicity (GRAVY). We used the Plant-mPLoc (http://www.csbio.sjtu.edu.cn/bioinf/plant-multi/, accessed on 14 December 2022) online tool to predict the subcellular localization of members of the *LcCCO* gene family.

### 4.2. Phylogeny and Sequence Alignment

The ClustalW program of MEGA-X [47] software was used to perform the multiple sequence alignment of *CCO* gene family protein sequences, which were obtained from nine species, including *L. chinense*, *Arabidopsis thaliana*, *Vitis vinifera*, *Solanum lycopersicum*, *Oryza sativa*, *Sorghum bicolor*, *Zea mays*, *Cinnamomum kanehirae* and *Amborella trichopoda*. The CCO protein IDs of each species are listed in Supplementary Table S1, respectively. The neighbor-joining method was used to construct the phylogenetic tree, in which 1000 replicates of bootstrap parameters were set. The online website iTOL (https://itol.embl.de/, accessed on 20 December 2022) was used to beautify and visualize the phylogenetic tree.

### 4.3. Gene Structure, Conserved Motif Analyses of CCO Genes

The intron-exon organization information of the *CCO* gene family was obtained from genomic annotation data (including *L. chinense*, *Arabidopsis thaliana*, V*itis vinifera, Solanum lycopersicum*, *Oryza sativa*, *Sorghum bicolor*, *Zea mays, Cinnamomum kanehirae* and *Amborella trichopoda*). The conserved motifs were predicted with MEME (https://meme-suite.org/meme/tools/meme, accessed on 16 December 2022), and the parameter settings were as follows: number of repetitions “any”, maximum motif number “20”, motif length “6-200”, and retaining the default values for other parameters. The gene structure and motifs were constructed and visualized using TBtools (https://github.com/CJ-Chen/TBtools, accessed on 16 December 2022).

### 4.4. Cis-Element Analysis in the Promoter of LcCCO Genes

The 2000 bp upstream DNA sequence of the initiation codon was extracted from the *L. chinense* genome file. The sequence was submitted to the PlantCARE (https://bioinformatics.psb.ugent.be/webtools/plantcare/html/, accessed on 16 December 2022) website for the analysis of *cis*-acting elements within the promoter region and visualized using TBtools.

### 4.5. Transcriptome Sequencing Analysis of LcCCO Gene Expression Levels in Different Organs, Somatic Embryogenesis and Multiple Stresses

In this study, transcriptome analysis was used to analyze the expression patterns of *LcCCO* family genes in different organs and somatic embryogenesis stages and under stress treatments. Transcriptome data of different organs of *L. chinense* and those of hybrid *Liriodendron* under high-temperature (40 °C) and drought stress at 0 h, 1 h, 3 h, 6 h, 12 h, 24 h and 72 h were downloaded from the NCBI website (the search numbers for the transcriptome raw data are arranged in Supplementary Table S2). The 4 °C low-temperature stress (0 h, 12 h, 24 h, and 48 h) and the hybrid *Liriodendron* somatic embryogenesis transcriptome data are data unpublished by the laboratory. For transcriptome sequencing under low-temperature stress, 3-month-old somatic embryo seedlings were selected for low-temperature (4 °C) treatment, and samples were collected according to different treatment durations for transcriptome sequencing. An embryogenic callus (PEM) was induced from immature embryos of hybrid *Liriodendron* seeds. Embryonic cells and tissues (E1~E4) were selected by suspension culture using a synchronous method and collected during the transformation phase (about every 3 days) and then germinated on a solid induction medium in a light-controlled growth chamber (E5~PL) [48]. The process of somatic embryogenesis was divided into 11 stages, including PEM (embryogenic callus), ES1 (liquid culture for 10 days), ES2 (screening culture for 2 days), ES3 (after 1 day of abscisic acid induction culture), ES4 (after 3 days of abscisic acid induction culture), ES5 (globular embryo stage), ES6 (heart-shaped embryo stage), ES7 (torpedo embryo stage), ES8 (immature cotyledon embryo), ES9 (mature cotyledon embryo) embryo) and PL (plantlet formation). The transcriptome sequencing was performed using each stage of somatic embryogenesis. Five expression matrices of *LcCCO* family genes in organs, somatic embryogenesis, and high-temperature, low-temperature and drought stress were obtained from the above transcriptome data and are shown in Supplementary Table S2.

### 4.6. Plant Material Treatments and qRT-PCR Analysis

Somatic embryo seedlings of 3 months old were cultured in an incubator (16 h light, 8 h dark) on 3/4 MS medium. The leaves and roots were harvested from three biological replicates of somatic seedlings after being treated separately with 20% polyethylene-glycol (PEG)-6000 solution and low temperature (4 °C) at 0, 6, 12, 24, and 48 h. Quantitative RT-PCR analysis was used to confirm the expression patterns of *LcNCED3a/b* in leaves and roots under the different treatments. Total RNA extraction was performed using an Eastep^®^ Super Total RNA Extraction Kit, Beijing, China (Promega, LS1040). First-strand cDNA was synthesized with a HiScript III 1st Strand cDNA Synthesis Kit (+gDNA wiper). The AceQ^®^ qPCR SYBR Green Master Mix (Without ROX) with 10 µL of Green Master Mix in a 20 µL reaction volume was used in Roche LightCycler^®^ 480 Real-Time PCR System to identify the *LcNCED3b* gene expression patterns. The reverse transcription and qRT-PCR kits were from Vazyme Biotech Co., Ltd., Nanjing, China. The qRT-PCR primers were designed using the online website primer3 (https://primer3.ut.ee/, accessed on 17 March 2023) and are listed in Supplementary Table S3. The relative expression of *LcNCED3b* was calculated by the 2^−∆∆CT^ method [49].

## 5. Conclusions

In summary, 10 *LcCCO* genes were identified based on *L. chinense* genome data, and a series of bioinformatics analysis and transcriptional expression analysis was conducted. Chromosomal localization analysis showed that the *LcCCO* gene was unevenly distributed on five chromosomes. Phylogenetic analysis showed that *LcCCO* gene family can be divided into five subfamilies: *NCED*, *CCD1*, *CCD4*, *CCD7*, *CCD8* and *CCDL*. The motif distribution and arrangement of *CCO* members in the same subfamily are similar, and collinearity analysis showed that *LcCCOs* and *LtCCOs* have a close evolutionary relationship. The promoter region of *LcCCO* family genes is widely distributed with *cis*-elements related to plant hormones, stress response and light response. Expression analysis showed that only the *LcCCD1b* gene was dominant in seven organs, the *LcCCD1a*/*LcCCD1b* gene was expressed throughout somatic embryogenesis, and *LcNCED3b* was highly expressed in the ES9 and PL stages. In addition, the expression of *LcCCD1b* was affected by high-temperature, low-temperature and drought stress, while *LcCCD7*/*8* and *LcCCDLa*/*b* were not expressed under the three kinds of stress. This study can provide reference for further studies of *LcCCO* family genes involving breeding and the stress-response function. The qRT-PCR analysis showed that the expression of *LcNCED3b* was first up-regulated and then down-regulated in both roots and leaves under drought stress, while the expression level of *LCNCED3b* under cold stress was very low.

## Figures and Tables

**Table 1:** Basic characteristics of 10 *LcCCO* genes in *L. chinense*.

Gene ID	Gene Name	Locus	CDS/bp	Size/aa	pI	Mr (kDa)	Subcellular Location
Lchi23418	*LcCCD4b*	Chr18	1743	580	6.58	64.2	Chloroplast
Lchi06122	*LcCCD1b*	Chr1	1062	353	6.22	40.3	Cytoplasm
Lchi06123	*LcCCD1a*	Chr1	1071	356	5.97	40.9	Cytoplasm
Lchi24696	*LcNCED3a*	Chr1	1554	517	6.22	56.8	Cytoplasm
Lchi08467	*LcCCD4a*	Chr8	1161	386	7.73	43.1	Cytoplasm
Lchi06042	*LcCCD7*	Chr8	1446	481	6.35	53.4	Chloroplast/Cytoplasm
Lchi30590	*LcNCED3b*	Chr19	1524	507	6.12	56.5	Cytoplasm
Lchi08901	*LcCCDLb*	Chr6	1056	351	6.42	39.8	Cytoplasm
Lchi08902	*LcCCDLa*	Chr6	1869	622	5.27	70.2	Cytoplasm
Lchi08924	*LcCCD8*	Chr6	1680	559	5.65	61.8	Cytoplasm

## Data Availability

Data sets supporting the conclusions and descriptions can be found in the manuscript and annexes. The complete data set used and analyzed in this study is available from the corresponding author upon reasonable request.

## Supplementary Materials

The following supporting information can be downloaded at: https://www.mdpi.com/article/10.3390/plants12101975/s1, Supplementary Table S1: The CCO protein/gene IDs of nine species, Gene structure and motif information, The location statistics of genes on chromosomes, The statistics of *cis*-acting elements, Statistical table of Ka/Ks values; Supplementary Table S2: The expression data of *LcCCO* genes in different organs, somatic embryogenesis and multiple stresses; Supplementary Table S3: The qRT-PCR data of *LcCCO* genes in root and leaf under cold and drought stresses.

## References

[1] Kloer D.P., Schulz G.E. (2006). Structural and biological aspects of carotenoid cleavage. Cell Mol. Life Sci..

[2] Schweiggert R.M., Carle R. (2017). Carotenoid deposition in plant and animal foods and its impact on bioavailability. Crit. Rev. Food Sci. Nutr..

[3] Amorim-Carrilho K.T., Cepeda A., Fente C., Regal P. (2014). Review of methods for analysis of carotenoids. TrAC.

[4] Niyogi K.K. (1999). Photoprotection revisited: Genetic and molecular approaches. Annu. Rev. Plant Physiol. Plant Mol. Biol..

[5] Nisar N., Li L., Lu S., Khin N.C., Pogson B.J. (2015). Carotenoid metabolism in plants. Mol. Plant.

[6] Ibdah M., Azulay Y., Portnoy V., Wasserman B., Bar E., Meir A., Burger Y., Hirschberg J., Schaffer A.A., Katzir N. (2006). Functional characterization of CmCCD1, a carotenoid cleavage dioxygenase from melon. Phytochemistry.

[7] Mellado-Ortega E., Hornero-Méndez D. (2015). Carotenoids in cereals: An ancient resource with present and future applications. Phytochem. Rev..

[8] Hao Z., Liu S., Hu L., Shi J., Chen J. (2020). Transcriptome analysis and metabolic profiling reveal the key role of carotenoids in the petal coloration of *Liriodendron tulipifera*. Hortic. Res..

[9] Moise A.R., von Lintig J., Palczewski K. (2005). Related enzymes solve evolutionarily recurrent problems in the metabolism of carotenoids. Trends Plant Sci..

[10] Hannoufa A., Hossain Z. (2012). Regulation of carotenoid accumulation in plants. Biocatal. Agric. Biotechnol..

[11] Huang F.C., Molnár P., Schwab W. (2009). Cloning and functional characterization of carotenoid cleavage dioxygenase 4 genes. J. Exp. Bot..

[12] Auldridge M.E., Block A., Vogel J.T., Dabney-Smith C., Mila I., Bouzayen M., Magallanes-Lundback M., DellaPenna D., McCarty D.R., Klee H.J. (2006). Characterization of three members of the Arabidopsis carotenoid cleavage dioxygenase family demonstrates the divergent roles of this multifunctional enzyme family. Plant J..

[13] Vallabhaneni R., Bradbury L.M., Wurtzel E.T. (2010). The carotenoid dioxygenase gene family in maize, sorghum, and rice. Arch. Biochem. Biophys..

[14] Walter M.H., Strack D. (2011). Carotenoids and their cleavage products: Biosynthesis and functions. Nat. Prod. Rep..

[15] Su W., Zhang C., Feng J., Feng A., You C., Ren Y., Wang D., Sun T., Su Y., Xu L. (2021). Genome-wide identification, characterization and expression analysis of the carotenoid cleavage oxygenase (CCO) gene family in *Saccharum*. Plant Physiol. Biochem..

[16] Kim Y., Hwang I., Jung H.J., Park J.I., Kang J.G., Nou I.S. (2016). Genome-wide classification and abiotic stress-responsive expression profiling of carotenoid oxygenase genes in *Brassica rapa* and *Brassica oleracea*. J. Plant Growth Regul..

[17] Schwartz S.H., Tan B.C., Gage D.A., Zeevaart J.A., McCarty D.R. (1997). Specific oxidative cleavage of carotenoids by VP14 of maize. Science.

[18] Chen H., Zuo X., Shao H., Fan S., Ma J., Zhang D., Zhao C., Yan X., Liu X., Han M. (2018). Genome-wide analysis of carotenoid cleavage oxygenase genes and their responses to various phytohormones and abiotic stresses in apple (*Malus domestica*). Plant Physiol. Biochem..

[19] Lashbrooke J.G., Young P.R., Dockrall S.J., Vasanth K., Vivier M.A. (2013). Functional characterisation of three members of the *Vitis vinifera* L. carotenoid cleavage dioxygenase gene family. BMC Plant Biol..

[20] Cheng D., Wang Z., Li S., Zhao J., Wei C., Zhang Y. (2022). Genome-wide identification of CCD gene family in six Cucurbitaceae species and its expression profiles in melon. Genes.

[21] Wei H., Movahedi A., Liu G., Li Y., Liu S., Yu C., Chen Y., Zhong F., Zhang J. (2022). Comprehensive Analysis of Carotenoid Cleavage Dioxygenases Gene Family and Its Expression in Response to Abiotic Stress in Poplar. Int. J. Mol. Sci..

[22] Yue X.Q., Zhang Y., Yang C.K., Li J.G., Rui X., Ding F., Hu F.C., Wang X.H., Ma W.Q., Zhou K.B. (2022). Genome-wide identification and expression analysis of carotenoid cleavage oxygenase genes in Litchi (*Litchi chinensis* Sonn.). BMC Plant Biol..

[23] Iuchi S., Kobayashi M., Taji T., Naramoto M., Seki M., Kato T., Tabata S., Kakubari Y., Yamaguchi-Shinozaki K., Shinozaki K. (2001). Regulation of drought tolerance by gene manipulation of 9-cis-epoxycarotenoid dioxygenase, a key enzyme in abscisic acid biosynthesis in *Arabidopsis*. Plant J..

[24] Wei Y., Wan H., Wu Z., Wang R., Ruan M., Ye Q., Li Z., Zhou G., Yao Z., Yang Y. (2016). A comprehensive analysis of carotenoid cleavage dioxygenases genes in *Solanum lycopersicum*. Plant Mol. Biol. Rep..

[25] Serra S. (2015). Recent advances in the synthesis of carotenoid-derived flavours and fragrances. Molecules.

[26] Ureshino K., Nakayama M., Miyajima I. (2016). Contribution made by the carotenoid cleavage dioxygenase 4 gene to yellow colour fade in azalea petals. Euphytica.

[27] Gomez-Roldan V., Fermas S., Brewer P.B., Puech-Pagès V., Dun E.A., Pillot J.P., Letisse F., Matusova R., Danoun S., Portais J.C. (2008). Strigolactone inhibition of shoot branching. Nature.

[28] Umehara M., Hanada A., Yoshida S., Akiyama K., Arite T., Takeda-Kamiya N., Magome H., Kamiya Y., Shirasu K., Yoneyama K. (2008). Inhibition of shoot branching by new terpenoid plant hormones. Nature.

[29] Gao J., Zhang T., Xu B., Jia L., Xiao B., Liu H., Liu L., Yan H., Xia Q. (2018). CRISPR/Cas9-Mediated Mutagenesis of Carotenoid Cleavage Dioxygenase 8 (CCD8) in Tobacco Affects Shoot and Root Architecture. Int. J. Mol. Sci..

[30] Alder A., Jamil M., Marzorati M., Bruno M., Vermathen M., Bigler P., Ghisla S., Bouwmeester H., Beyer P., Al-Babili S. (2012). The path from β-carotene to carlactone, a strigolactone-like plant hormone. Science.

[31] Ruyter-Spira C., Al-Babili S., Van Der Krol S., Bouwmeester H. (2013). The biology of strigolactones. Trends Plant Sci..

[32] Morris S.E., Turnbull C.G., Murfet I.C., Beveridge C.A. (2001). Mutational analysis of branching in pea. Evidence that Rms1 and Rms5 regulate the same novel signal. Plant Physiol..

[33] Koltai H. (2011). Strigolactones are regulators of root development. New Phytol..

[34] Decker E.L., Alder A., Hunn S., Ferguson J., Lehtonen M.T., Scheler B., Kerres K.L., Wiedemann G., Safavi-Rizi V., Nordzieke S. (2017). Strigolactone biosynthesis is evolutionarily conserved, regulated by phosphate starvation and contributes to resistance against phytopathogenic fungi in a moss, *Physcomitrella patens*. New Phytol..

[35] Torres-Vera R., García J.M., Pozo M.J., López-Ráez J.A. (2014). Do strigolactones contribute to plant defence?. Mol. Plant Pathol..

[36] Qin X., Zeevaart J.A. (2002). Overexpression of a 9-cis-epoxycarotenoid dioxygenase gene in Nicotiana plumbaginifolia increases abscisic acid and phaseic acid levels and enhances drought tolerance. Plant Physiol..

[37] Chen J., Hao Z., Guang X., Zhao C., Wang P., Xue L., Zhu Q., Yang L., Sheng Y., Zhou Y. (2019). Liriodendron genome sheds light on angiosperm phylogeny and species-pair differentiation. Nat. Plants.

[38] Duraisamy G.S., Mishra A.K., Kocabek T., Matoušek J. (2016). Identification and characterization of promoters and cis-regulatory elements of genes involved in secondary metabolites production in hop (*Humulus lupulus* L.). Comput. Biol. Chem..

[39] Waadt R., Seller C.A., Hsu P.K., Takahashi Y., Munemasa S., Schroeder J.I. (2022). Plant hormone regulation of abiotic stress responses. Nat. Rev. Mol. Cell Biol..

[40] González-Verdejo C.I., Obrero Á., Román B., Gómez P. (2015). Expression Profile of Carotenoid Cleavage Dioxygenase Genes in Summer Squash (*Cucurbita pepo* L.). Plant Foods Hum. Nutr..

[41] Walter M.H., Floss D.S., Strack D. (2010). Apocarotenoids: Hormones, mycorrhizal metabolites and aroma volatiles. Planta.

[42] Espasandin F.D., Maiale S.J., Calzadilla P., Ruiz O.A., Sansberro P.A. (2014). Transcriptional regulation of 9-cis-epoxycarotenoid dioxygenase (NCED) gene by putrescine accumulation positively modulates ABA synthesis and drought tolerance in Lotus tenuis plants. Plant Physiol. Biochem..

[43] Zhang X.H., Liu H.Q., Guo Q.W., Zheng C.F., Li C.S., Xiang X.M., Zhao D.F., Liu J., Luo J., Zhao D.K. (2016). Genome-wide identification, phylogenetic relationships, and expression analysis of the carotenoid cleavage oxygenase gene family in pepper. Genet. Mol. Res..

[44] Shekhawat K., Almeida-Trapp M., García-Ramírez G.X., Hirt H. (2022). Beat the heat: Plant- and microbe-mediated strategies for crop thermotolerance. Trends Plant Sci..

[45] Nakashima K., Yamaguchi-Shinozaki K. (2013). ABA signaling in stress-response and seed development. Plant Cell Rep..

[46] Eddy S.R. (1996). Hidden markov models. Curr. Opin. Struct. Biol..

[47] Kumar S., Stecher G., Li M., Knyaz C., Tamura K. (2018). MEGA X: Molecular Evolutionary Genetics Analysis across Computing Platforms. Mol. Biol. Evol..

[48] Li T., Chen J., Qiu S., Zhang Y., Wang P., Yang L., Lu Y., Shi J. (2012). Deep sequencing and microarray hybridization identify conserved and species-specific microRNAs during somatic embryogenesis in hybrid yellow poplar. PLoS ONE.

[49] Livak K.J., Schmittgen T.D. (2001). Analysis of relative gene expression data using real-time quantitative PCR and the 2^−ΔΔCT^ method. Methods.

